# A Bayesian gene network reveals insight into the JAK-STAT pathway in systemic lupus erythematosus

**DOI:** 10.1371/journal.pone.0225651

**Published:** 2019-12-02

**Authors:** Yupeng Li, Richard E. Higgs, Robert W. Hoffman, Ernst R. Dow, Xiong Liu, Michelle Petri, Daniel J. Wallace, Thomas Dörner, Brian J. Eastwood, Bradley B. Miller, Yushi Liu

**Affiliations:** 1 Eli Lilly and Company, Indianapolis, Indiana, United States of America; 2 Hopkins Lupus Center, John Hopkins University, Baltimore, Maryland, United States of America; 3 David Geffen School of Medicine, University of California Los Angeles, Los Angeles, California, United States of America; 4 Charité University Hospitals, Berlin, Germany; Texas A&M University, UNITED STATES

## Abstract

Systemic lupus erythematosus (SLE) is a chronic, remitting, and relapsing, inflammatory disease involving multiple organs, which exhibits abnormalities of both the innate and adaptive immune responses. A limited number of transcriptomic studies have characterized the gene pathways involved in SLE in an attempt to identify the key pathogenic drivers of the disease. In order to further advance our understanding of the pathogenesis of SLE, we used a novel Bayesian network algorithm to hybridize knowledge- and data-driven methods, and then applied the algorithm to build an SLE gene network using transcriptomic data from 1,760 SLE patients’ RNA from the two tabalumab Phase III trials (ILLUMINATE-I & -II), the largest SLE RNA dataset to date. Further, based on the gene network, we carried out hub- and key driver-gene analyses for gene prioritization. Our analyses identified that the JAK-STAT pathway genes, including *JAK2*, *STAT1*, and *STAT2*, played essential roles in SLE pathogenesis, and reaffirmed the recent discovery of pathogenic relevance of JAK-STAT signaling in SLE. Additionally, we showed that other genes, such as *IRF1*, *IRF7*, *PDIA4*, *FAM72C*, *TNFSF10*, *DHX58*, *SIGLEC1*, and *PML*, may be also important in SLE and serve as potential therapeutic targets for SLE. In summary, using a hybridized network construction approach, we systematically investigated gene-gene interactions based on their transcriptomic profiles, prioritized genes based on their importance in the network structure, and revealed new insights into SLE activity.

## Introduction

SLE is a prototypic, systemic autoimmune disease characterized by inflammation of multiple organ systems and dysregulation of both innate and adaptive immunity. Genetic studies have identified more than 80 SLE-associated gene loci that contribute to disease susceptibility [1]. After decades of many unsuccessful trials, several new drugs have reported success in Phase II and III clinical trials of lupus. However, despite this success, the central drivers and key molecular targets that drive the activity of SLE remain unclear [2–5].

Using gene expression profiling, the cytokine class of type I interferon (IFN) responsive genes was shown to be elevated in SLE. These genes are collectively known as the IFN response gene signature and comprise a large group of IFN proteins that regulate the function of immune system. Subsequently, this led to clinical trials that focused on characterizing the IFN signature [6]. Approximately 50% to 75% of SLE patients enrolled in industry-sponsored Phase II and III SLE trials that characterized IFN responsive gene profiles were found to be type I IFN-signature positive (IFN-positive) [7–9]. The high prevalence of the IFN signature in SLE, particularly among clinical trial patients, supported the concept that identification of subgroups of patients with high IFN gene expression and targeting type I IFN could be an effective strategy for the treatment of SLE. However, Phase II and III studies targeting type I IFN demonstrated variable results [7, 10]. These results instigated research to identify new and more relevant targets for drug therapy.

Statistical methods have been developed to infer gene-gene interactions (GGI) and gene regulatory relationships using large amounts of transcriptomic data. Such inferred GGIs can be represented by a graph or network, in which nodes are genes and edges represent GGIs. Rather than providing a list of putative disease-relevant genes, a gene network approach provides a systematic view of the interplay between multiple genes and pathways, helps to understand how genes contribute to disease pathogenesis, and prioritizes key driver genes for drug development. There are three popular gene network construction methods [11]. The most popular method is correlation-based (also known as co-expression-based), which uses correlation to connect genes [12]. However, the drawback of this method is the inability to distinguish direct and indirect interactions. In practice, this method is often used as a clustering method to identify modules of genes with similar expression profiles. The second method is the Markov method, which is based on conditional dependence detection to describe a set of random variables with Markov properties. In theory, the Markov property ensures only direct connections remain in the network. The third method is the Bayesian method, which describes a set of random variables using their conditional dependence in an acyclic directed graph. The edges in a Bayesian network are directed, and under proper assumptions, can be viewed as causal relationships [13]. Despite the appealing theoretical properties, the Bayesian network method is challenging with limited sample sizes because the number of possible network topologies increases super-exponentially with the number of genes.

In this study, we developed a novel computational method to construct a Bayesian gene network to tackle the high-dimensionality issue and applied this method to SLE. Although type I INFs have been implicated in the pathogenesis of SLE, our Bayesian gene network revealed that some JAK-STAT pathway genes (e.g. *JAK2*, *STAT1*, and *STAT2*) may also be central to SLE. This is consistent with the recent discovery of the pathogenic and therapeutic relevance of JAK-STAT signaling in SLE [14, 15]. Our network construction and analysis in SLE demonstrated the value of these genes in the understanding of the disease and in the identification of new therapeutic targets.

## Materials and methods

### Gene expression data

**Fig 1:**
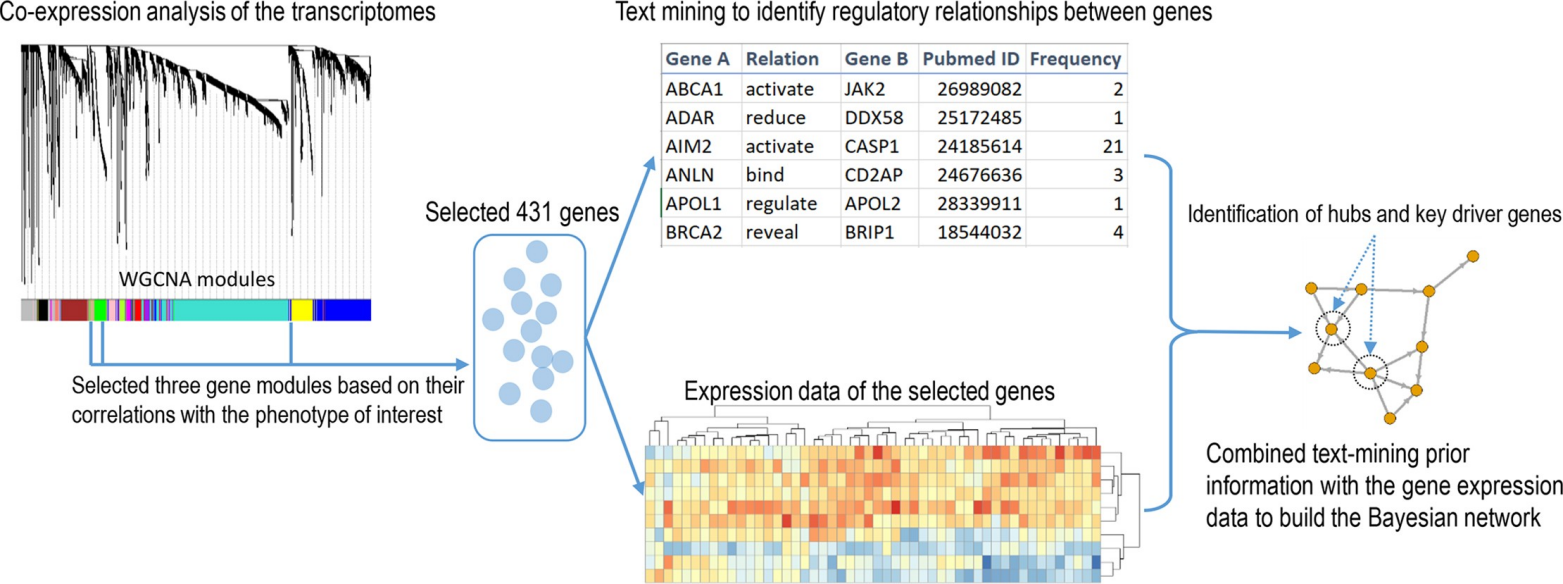
Overall analysis workflow.

Gene expression data were obtained from 1,760 SLE patients in two Phase III, 52-week, randomized, placebo-controlled, double-blind studies (ILLUMINATE-1 and -2) [16, 17]. Blood samples were collected in Tempus tubes (Thermo Fisher Scientific) at baseline, Week 16, and Week 52. Transcriptomic results for each individual sample were analyzed using the Affymetrix Human Transcriptome Array 2.0 (HTA2). Other details of transcriptomic data collection, processing, and statistical analysis have been previously described [7]. The dataset is publicly available in the Gene Expression Omnibus repository (accession IDs: GSE88884 and GSE88887). For our analysis, only baseline transcriptomic data from 1,760 SLE patients were used for network construction. Additionally, the results of analyses to test differential expression (SLE vs. healthy controls) were used as additional input for key driver gene analysis. The overall analysis workflow for candidate gene selection, gene network construction, and analysis is illustrated in Fig 1.

### Candidate gene selection

A co-expression network using all SLE patient baseline samples was built and has been previously described [7]. Briefly, transcripts with a coefficient of variation less than 25% were considered as ‘uninformative’ and removed. Five samples were removed based on weighted gene co-expression network analysis (WGCNA) outlier detection [12]. The remaining transcripts were clustered into 14 co-expression modules using WGCNA [7, 12]. The IFN signature, the first principal component derived from 34 of the 164 pre-selected IFN response genes that were the highest covariates, was an independent predictor of time to severe flare events [7]. As we were interested to investigate the function of these genes in relation to both IFN signature and disease flare, we selected three gene modules (Tan, Yellow, and Green) from the co-expression network [7] as candidates for the following Bayesian gene network construction. The expression of these three gene modules significantly correlated with the IFN signature and was associated with time to disease flare (Table 1).

**Table 1:** Three selected WGCNA modules.

Module	Gene (transcript cluster) number	Correlation with IFN signature (P-value)	Association with time to flare*	Top 5 enriched gene ontology terms	False discovery rate of the enrichment test
Yellow	229 (264)	0.90 (0.00E+00)	1.00E-05	cellular response to type I interferon	1.16E-32
				response to type I interferon	1.16E-32
				type I interferon signaling pathway	1.13E-31
				defense response to virus	8.43E-26
				defense response	1.40E-24
Tan	64 (64)	0.42 (3.00E-48)	0.00E+00	nuclear division	4.23E-23
				organelle fission	4.43E-23
				cell cycle	6.20E-23
				mitotic cell cycle	2.79E-22
				mitotic nuclear division	7.84E-22
Green	138 (138)	0.34 (3.00E-48)	0.00E+00	humoral immune response	1.48E-06
				complement activation, classical pathway	1.58E-06
				humoral immune response mediated by circulating immunoglobulin	2.28E-06
				immune response-regulating cell surface receptor signaling pathway involved in phagocytosis	2.28E-06
				Fc-gamma receptor signaling pathway involved in phagocytosis	2.28E-06

* The association was measured by Cox proportional hazards model *λ*(*t*|*x_i_*) = *λ*_0_(*t*) exp(*x_i_*⋅*β*), and P-values from the models were shown in the table.

### Prior information

Prior information of GGIs can be obtained using multiple methods (e.g. text-mining, existing pathway databases, protein-protein interactions, and genetic interactions). Here we used text-mining to retrieve gene regulation information from the literature using I2E [18]. There is a vast corpus of molecular biology literature available in MEDLINE (PubMed), and I2E utilizes advanced natural language processing techniques, such as information extraction, to process abstracts from MEDLINE and produce a structured representation of the knowledge of interest. Specifically, we used the gene ontologies in I2E to find all relevant synonyms for a gene and used a comprehensive biological relationship query to find all available gene-gene relationships. This I2E search strategy provided a rigorous and extensive analysis of MEDLINE to identify high-quality gene-regulation relationships. The species, disease, tissue, and cell type were not restricted in the search query. The GGIs retrieved from I2E can be represented as a prior network, in which nodes are genes and directed edges represent their potential regulatory relationships. Multiple documents may describe the same GGI, and we used the number of documents to score the strength of GGIs. Therefore, the number of documents describing the same GGI was assigned as edge weight in the prior network, representing the confidence of the prior information.

### Bayesian network construction algorithm

**Fig 2:**
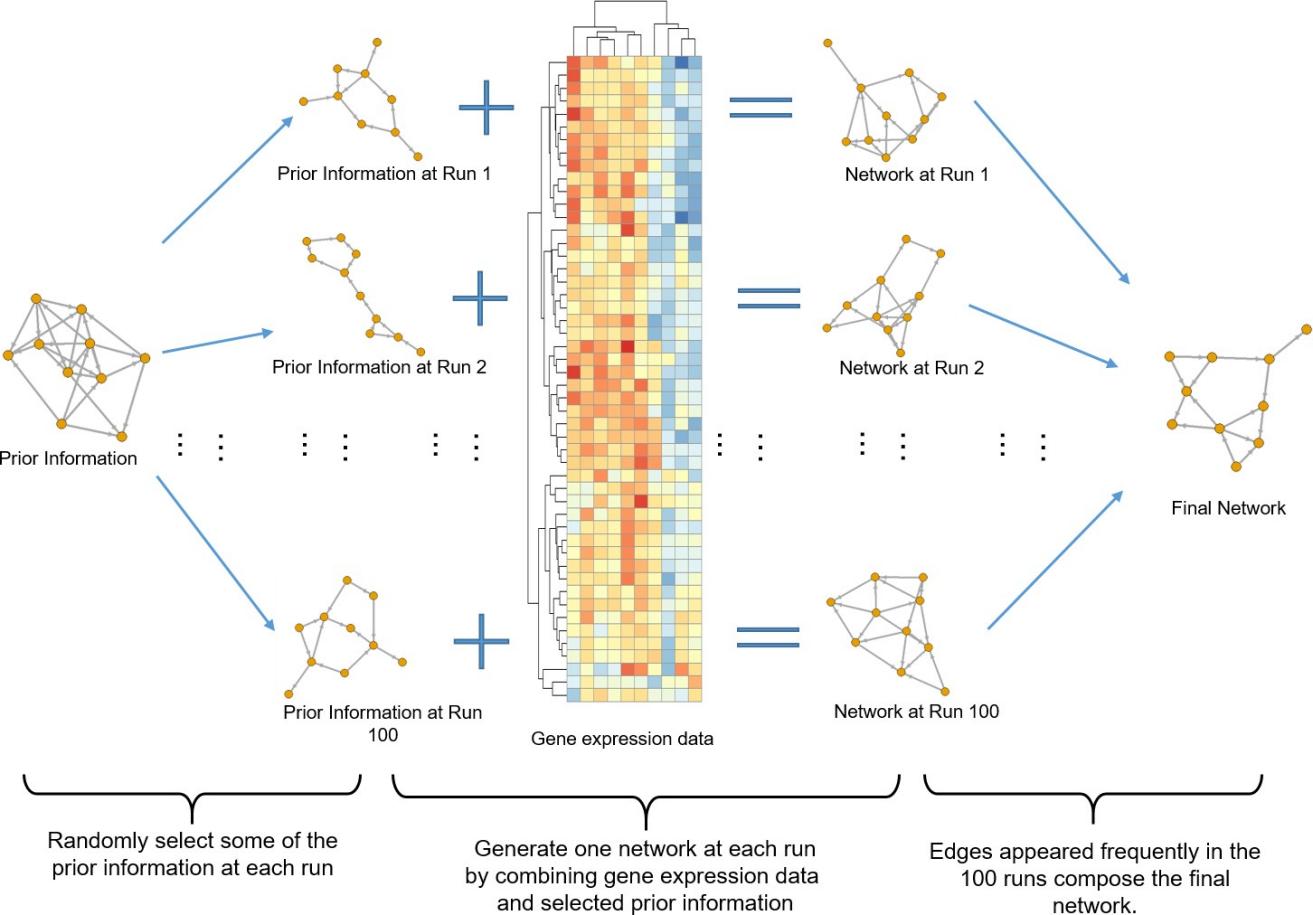
Algorithm workflow.

With a limited sample size, structure learning in a Bayesian network is a challenging high-dimension problem. Motivated by Zhang *et al*’s work [19], we proposed a flexible approach to utilize known information as prior information, and integrate it with transcriptome data to generate the potential causal network. Here, the prior information was derived from text-mining results and it was integrated with the transcriptomic data of baseline patients from the ILLUMINATE-1 and -2 trials. The algorithm is illustrated in Fig 2 and described as follows.

1. Prior edges (i.e. GGIs from text-mining) were randomly sampled based on the text-mining results. The selection probability of each edge in the prior network was based on exponential distribution, 0.85(1−*e^−x^*) where *x* was the edge weight (i.e. the number of documents that supported the GGI). Because the prior network contained a few loops and bi-directional edges, once a set of edges was selected, this ‘mini prior network’ was further pruned to generate a directed acyclic graph, in which the feedback arc set (i.e. the bi-directional or loop-forming edges) was removed. The minimum feedback arc set, which had minimal total weight among all possible feedback arc sets, was removed using the integer programming algorithm implemented by the *igraph* package [20].
2. A network was built based on the gene expression data using R package *bnlearn* [21], but keeping the selected prior edges in *Step 1* in the network structure. The learned structure would include two types of edges: edges selected in *Step 1* and edges derived using the gene expression data. A score-based approach was used to learn the Bayesian network structure, which assigned each candidate structure a score that measured how well the structure describes the data and then found the structure that maximizes the score, formally expressed as max*_G,θ_ L*(〈*G,θ*〉;*D*), i.e. to find a graph structure *G* with parameter *θ* that maximizes the likelihood given the data set *D* [22]. Here, we used the hill-climbing algorithm. It was a score-based heuristic search algorithm to iteratively perform a single-edge change for attempting to find a higher score at each step.

The two steps shown above were repeated 100 times.

Once the 100 runs were finished, edges from all runs were aggregated and counted. The frequency range for all the edges was integers from 1 to 100 and defined as ‘aggregated weight’. The edges with high frequency were considered stable and reliable interactions and vice versa. Subsequently, a reliability cutoff would be needed to filter out low weight edges to generate the final network. Many real-world networks (e.g. social network, the worldwide web, airline network, protein-protein interaction network) are scale-free [23], which means the node degrees follow a power-law distribution. Therefore, we used the scale-free topology criterion [12] to select the reliability cutoff. At each cutoff, the degrees of nodes were fitted to a power-law distribution using a linear model after log transformation. *R*^2^ from the fitted linear model was considered as a measure of scale-free topology. Usually, the *R*^2^ increases when the cutoff increases. The cutoff was chosen once the *R*^2^ first achieved above 0.8.

### Simulation

Simulations were carried out to test the influence of prior information on the accuracy and variability of network construction. We considered a 46-gene network (http://www.bnlearn.com/bnrepository/gaussian-medium.html) as the ‘true’ network structure, and generated gene expression data with 300 samples using the forward sampling method in *bnlearn* [21]. We also simulated multiple sets of prior information with different precisions (e.g. 0.8, 0.6, 0.4, and 0.2). For example, 0.8 precision meant the 80% prior edges were correct and 20% prior edges were wrong. The prior edge number was equal to 70, i.e. the edge number in the “true” network. We also tested the null prior (precision = 0), which meant the final network was totally data-driven. We repeated the algorithm 20 times for each precision value. At each time, the prior information was randomly generated based on the precision value.

### Key driver genes

Key driver genes, or master regulator genes are defined as those which have a significant effect on the expression of neighbor genes. Depending on which neighbors were included, we defined two types of key driver genes. First, key driver genes are genes whose ‘direct children’ tend to be differentially-expressed for SLE versus healthy controls. Second, key driver genes can be those whose ‘Markov blanket genes’ tend to be differentially-expressed genes. In a Bayesian network, the Markov blanket of a node includes its parents, children, and the other parents of its children. Mathematically, the rest of the network is conditionally independent of that node given the Markov blanket. Key driver genes were those genes which have not only relatively more neighbors, but also most of those neighbors are differentially-expressed in SLE versus healthy controls. We assumed key driver genes should have relatively more neighbor genes such that only the top 20 genes based on the number of neighbors, either children or Markov blanket, were selected for the key driver gene analyses. Then genes were ranked based on the average Z-scores of their neighbors; Z-scores were derived using the P-values from differential gene expression analysis.

## Results

### Simulation demonstrated the high stability and accuracy of the algorithm

Our algorithm utilized a model-averaging strategy and integrated the prior information in order to handle the high-dimensionality issue and to achieve high stability and accuracy. Due to high-dimensionality, the hill-climbing method is very sensitive to the start point and easily falls into local minima. Based on our algorithm, the final network results from aggregating and filtering of 100 individual networks. Only the common edges from the 100 individual networks are kept. This model-averaging method is a common practice to reduce the model variance [24]. Accurate prior information is able to restrict the search space or guide the search path to be close to the global minimum. Accurate prior information can increase both the accuracy and the stability of a model, and we used simulation to test the hypothesis. Twenty final networks were generated based on the simulated gene expression data and prior information at each prior precision setting, as described above.

**Fig 3:**
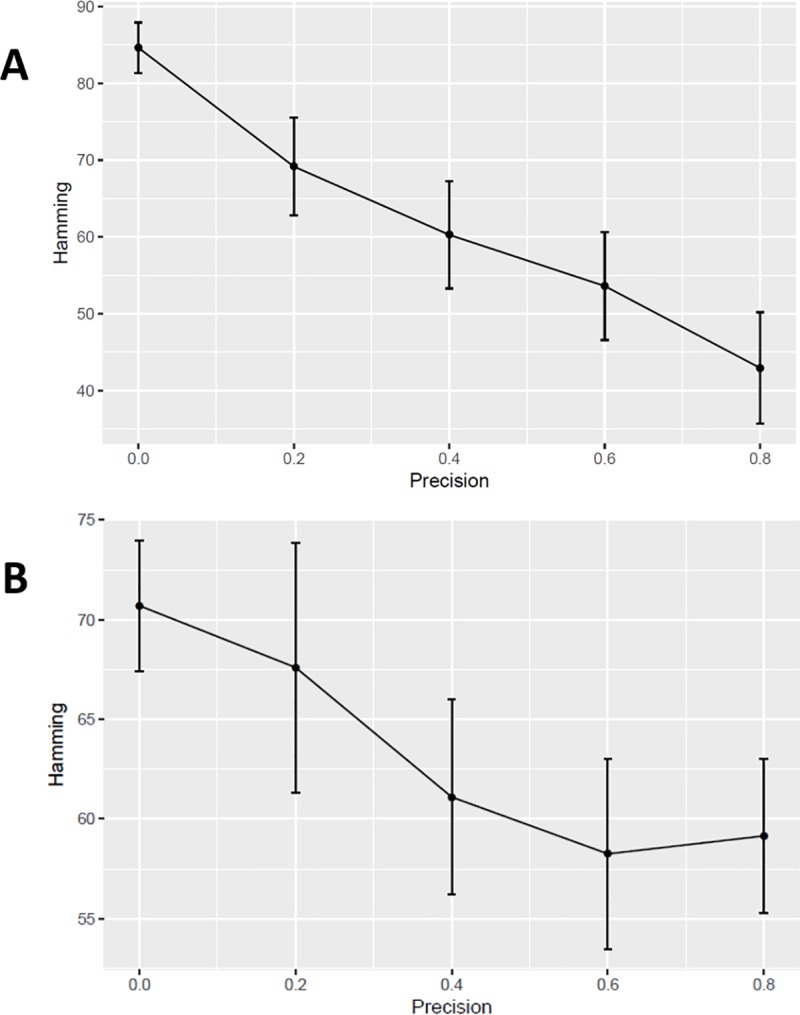
Stability and accuracy assessment using the simulation. The stability (A) is measured by the mean of Hamming distances between pairs of 100 individual networks in 100 runs for each final network. The accuracy (B) is measured by the Hamming distance between the synthetic true network and a predicted network. The simulation was repeated 20 times for each precision setting. Precision represents the accuracy of the prior information, except precision 0, which means no prior information. The error bar is +/-1 standard deviation.

If a model has low variance (i.e. high stability), the individual networks from 100 runs would be relatively similar. The similarity between two networks can be measured by Hamming distance, i.e. the number of addition/deletion operations required to turn the edge set of one network into that the other network. Therefore, we calculated the Hamming distances between pairs of the 100 individual networks in the 100 runs. The mean of the Hamming distances can be used to assess the stability and a lower value indicates higher stability, which means the 100 individual networks are generally more similar to each other. The stabilities of the networks without prior information were significantly lower than those with prior information, and more importantly, increasing the accuracy of prior led to higher network stability (Fig 3A). In addition, we used the Hamming distance between the true network and the generated network to assess the accuracy of the algorithm. A shorter distance meant more similarity between the two networks. As shown in Fig 3B, the accuracy of the generated network was significantly improved with more accurate prior information.

### Three WGCNA modules had significantly high correlations with IFN signature

We focused on the set of genes whose expression significantly associated with IFN signature and time to flare. Three WGCNA modules from the previous analysis, Yellow, Tan, and Green, met the criteria [7]. Based on a gene ontology enrichment test, these modules could be annotated as type I interferon module, cell cycle module, or humoral immune response module, respectively (Table 1). The IFN signature was calculated using the expression profiles of the 34 IFN response genes. The Yellow module had all the 34 genes and expectedly had the highest correlation with the IFN signature. 108 out of the 164 pre-defined IFN response genes were found the in Yellow module, while none were found in the Tan and Green modules. This suggested that factors other than the IFN signature contribute to the SLE disease activity. We were interested to identify the regulatory pathways among genes in the Yellow module and their crosstalk with genes in the Tan and Green modules. Therefore, we selected all the 466 transcript clusters and 431 corresponding genes in the three modules for the following Bayesian network analyses (S1 Table).

### Prior information

**Fig 4:**
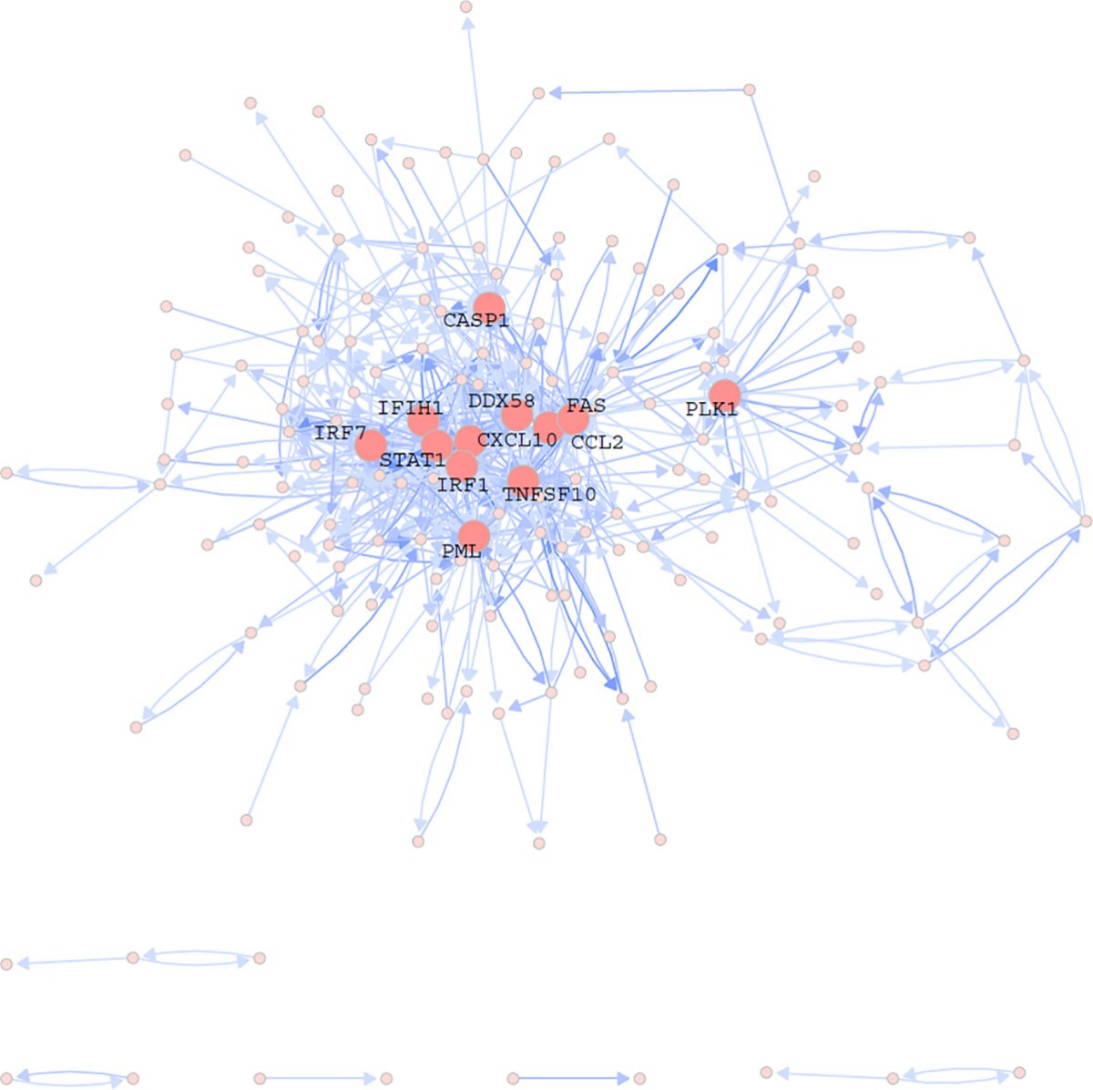
The prior network. The node represents the gene, the edge is the regulatory relationship between two genes, and the color darkness of the edge corresponds to the edge weight, i.e. the number of documents showing the regulatory relationship. The top 12 hub genes are highlighted.

17 histone cluster and 112 immunoglobulin genes were removed to reduce complexity. Removing these was not felt to impact the analysis due to the fact that their presence was interpreted to reflect the expression of house-keeping genes and high levels of autoantibody activity [7]. The remaining 302 genes were used to query the text-mining tool, I2E. We retrieved a total of 1,904 hits (S2 Table). Among the 1,904 hits, there were 595 distinct gene pair relationships. One unique gene pair relationship meant that gene A has an effect on gene B. The number of hits was recorded as the weight for that gene pair relationship. A prior network with 167 nodes and 595 edges excluding singletons was constructed using the text-mining result, in which nodes were genes and edges were unique gene pair relationships with assigned weights (Fig 4 and S3 Table).

### The Bayesian gene network

**Fig 5:**
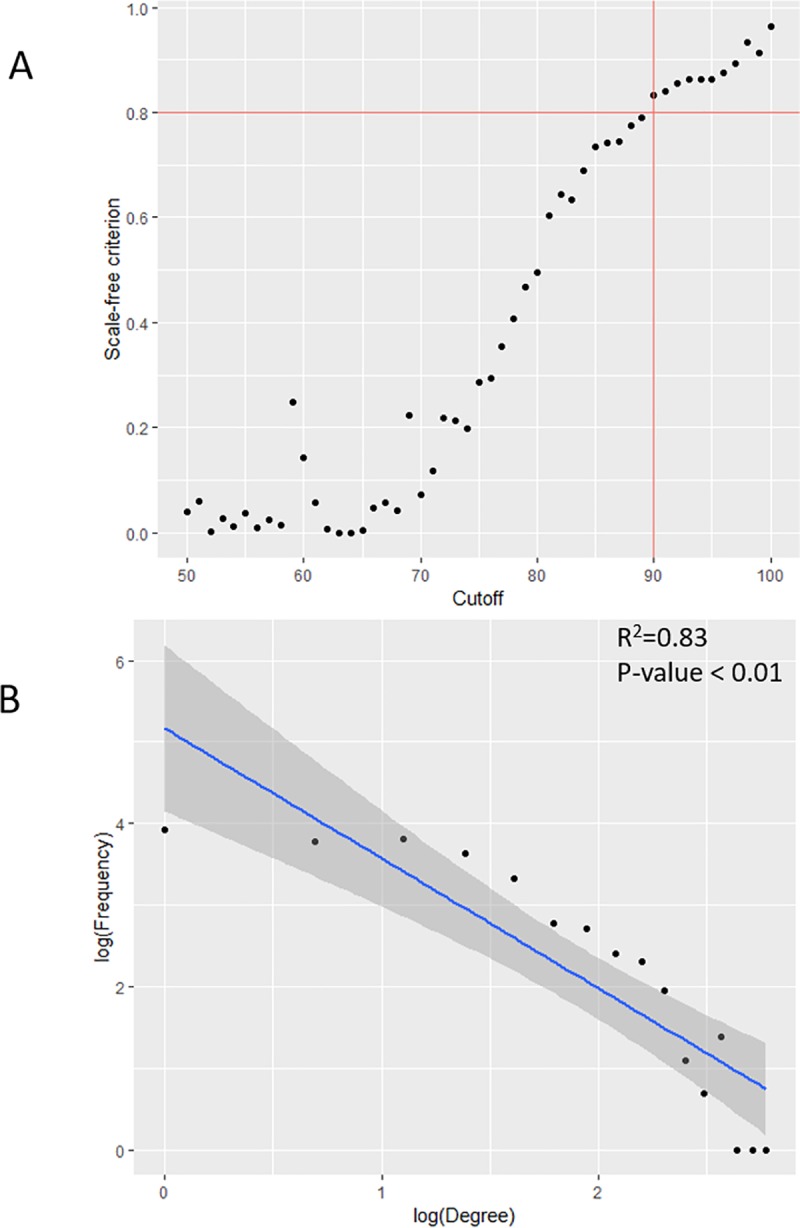
Reliability cutoff selection and the degree distribution of the final network. (A) The reliability cutoff selection was based on a scale-free criterion and the cutoff was set to 90, where the scale-free criterion first achieved above 0.8 when increasing the cutoff from 50 to 100. (B) For the degree distribution of the final network, the distribution was log-transformed to show it generally fitted the power-law distribution.

**Fig 6:**
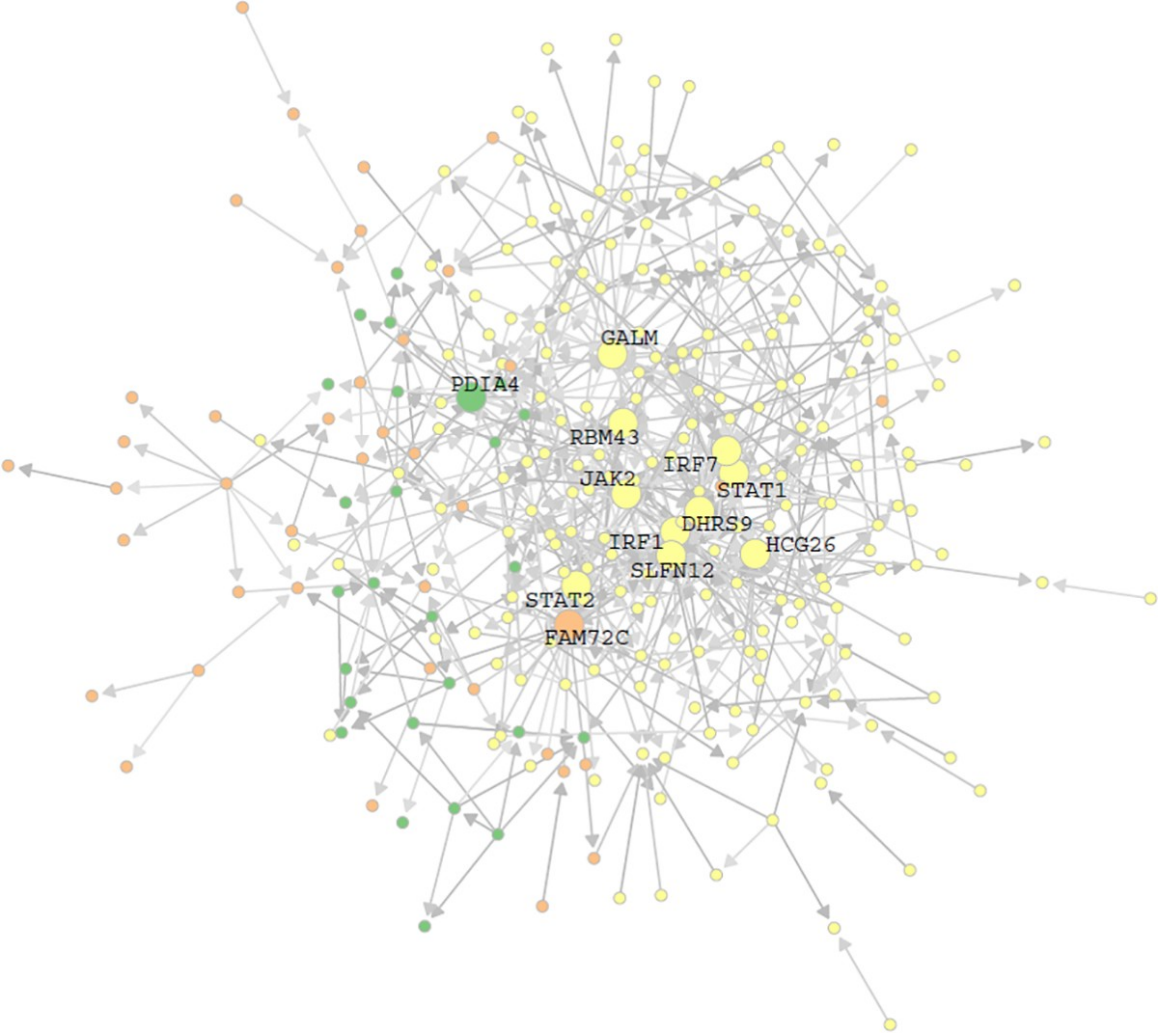
The final network. The node represents gene and the edge is the regulatory relationship between two genes. The color of a node represents one of the WGCNA modules, Yellow, Green or Tan, and the color darkness of the edge corresponds to the edge weight, i.e. the edge frequency in the 100 runs. The top 12 hub genes are highlighted.

The reliability cutoff was set to 90, as an edge should appear at least 90 times out of the 100 random runs. Usually, the network becomes sparser and more like a scale-free network when the reliability cutoff increases (Fig 5A). We selected the cutoff, 90, at which the network first achieved above a 0.8 scale-free criterion when increasing the reliability cutoff from 50 to 100 (Fig 5). The final network had 277 nodes and 598 edges, excluding singletons (Fig 6 and S4 Table).

During the network construction process, the probability that one prior edge was selected was based on the exponential distribution (1–*e^−x^*) multiplied by a ceiling parameter 0.85, where *x* was the edge weight (i.e. counts of documents supporting the GGI). A higher weight meant a higher probability of being selected at each run, and thus remaining in the final network. The selection probability ranged from 0.537 to close to 0.85 for each edge at each run (Table 2). These selection probabilities seemed high, but we used 90 as the reliability cutoff, such that any edge in the final network would need to be present in at least 90 out of 100 runs. Thus, most of the prior edges were highly unlikely to remain in the final network if they were random noise (Table 2). This was supported by the 50 prior edges that remained in the final network, which was much higher than expected based on binomial distribution (11.31 edges). At most weight levels, the numbers of prior edges remaining in the final network were significantly higher than expected (Table 2). This indicated that many of these prior edges were supported by both existing knowledge and gene expression data. Nevertheless, both the ceiling parameter 0.85 and the scale-free criterion of 0.8 were empirical settings. Further simulations and studies will be necessary to find a more systematic way to set the parameters.

**Table 2:** The impact of prior weight.

Prior weight	Number of prior edges	Selection probability per edge in one run^+^	Probability of one prior edge remaining in final network by chance^++^	Expected number of prior edges remaining in final network by chance	Number of prior edges remaining in final network	P-value
**1**	269	0.537	4.67E-15	1.26E-12	14	9.98E-178*
**2**	122	0.735	3.85E-05	4.69E-03	4	1.92E-11*
**3**	67	0.808	9.39E-03	6.29E-01	6	4.19E-05*
**4**	42	0.834	4.53E-02	1.90	10	1.41E-05*
**5**	20	0.844	7.53E-02	1.50	4	5.90E-02
**6**	16	0.848	8.99E-02	1.43	4	4.94E-02*
**7**	6	0.849	9.59E-02	5.75E-01	0	1
**> = 8**	53	≈0.850	≈9.81E-02	≈5.20	8	≈1.44E-01

^+^ Selection probability per edge in one run = 0.85(1-e—prior weight).

^++^ Assuming the reliability cutoff is 90, which means one prior network should be appear at least 90 times out of 100 runs. The probability is calculated based on binomial distribution.

* Statistical significance (P-value <0.05).

**Fig 7:**
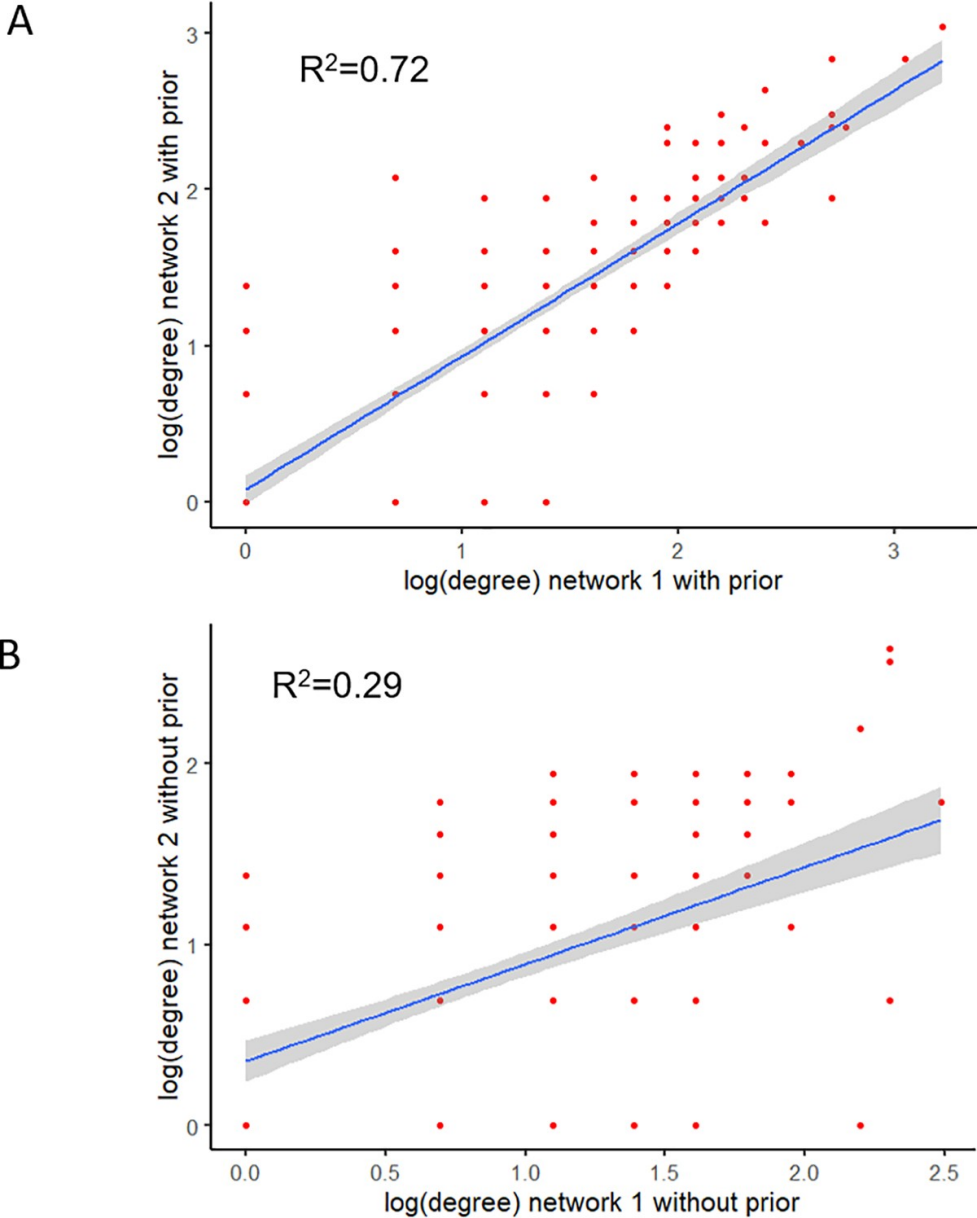
The relationship of node degrees for two networks. (A) with prior information and (B) without prior information.

As demonstrated by our simulations, the network without prior information had low stability and accuracy. Previously, we used the mean of Hamming distances between pairs of the 100 individual networks in the 100 runs to assess the stability of the networks. The value from the networks without prior information was 6962.83 ± 169.59, higher than that with prior information (6462.59 ± 176.02). It had 364 edges, which was also lower than that with prior information (598). The higher mean Hamming distance and fewer edges indicated the individual networks from 100 runs were quite different from each other if no prior information was provided, i.e. the model without prior information had low stability. Another way of assessing stability was to rerun the whole algorithm with the same setting and compare the two results (Fig 7). With prior information, the two networks had 598 and 536 edges, respectively, and had 467 overlapped edges (78.1% and 87.1% of edges overlap with each other, respectively). Without prior information, only 65.7% and 66.0% of edges overlapped with each other, respectively. The node degree distributions from two networks with prior information were more similar than those from two networks without prior information (Fig 7).

### Hub genes

The hub gene is an important concept in network analysis. It is defined as a gene that is over-connected compared with an ‘average’ gene. Such genes are important because knockdown of these genes will potentially perturb more genes or pathways. Thus, understanding of their functions could improve our understanding of the disease mechanism.

We compared the hub genes from the prior network and the final network in the setting of SLE pathogenesis. Given that the prior network was generated based only on current knowledge about GGIs, the hub genes in the network were biased towards genes that were of research interest. Although these genes were immune function-focused due to the selection criteria, they may not be SLE specific. Table 3 shows the lists of top hub genes from the prior network and the final network. As a comparison, *STAT1*, *IRF1*, and *IRF7* were overlapping genes, suggesting the importance of these well-studied genes in SLE; all three genes were critical in the type I interferon and JAK-STAT pathways [25]. In contrast to the prior network, the final network identified more JAK-STAT pathway genes (*JAK2* and *STAT2*).

**Table 3:** Top hub genes in prior and final networks.

Prior network*				Final network			
Gene	No. of direct neighbors	No. of children**	No. of parents**	Gene	No. of direct neighbors	No. of children	No. of parents
STAT1**	59	34	25	**IRF1**	25	25	0
**IRF1**	53	30	23	FAM72C	21	0	21
DDX58	40	17	23	PDIA4	16	15	1
FAS	38	11	27	JAK2	15	15	0
PML	32	21	11	SLFN12	15	1	14
CXCL10	31	11	20	GALM	15	1	14
TNFSF10	30	17	13	DHRS9	15	0	15
CCL2	28	8	20	**STAT1**	13	13	0
**IRF7**	28	15	13	HCG26	13	0	13
CASP1	25	10	15	STAT2	11	10	1
PLK1	24	13	11	**IRF7**	11	4	7
IFIH1	22	10	12	RBM43	11	0	11

Genes are sorted based on the number of direct neighbors

* Prior information includes bi-directed edges.

** The common genes in both lists are in bold.

**Fig 8:**
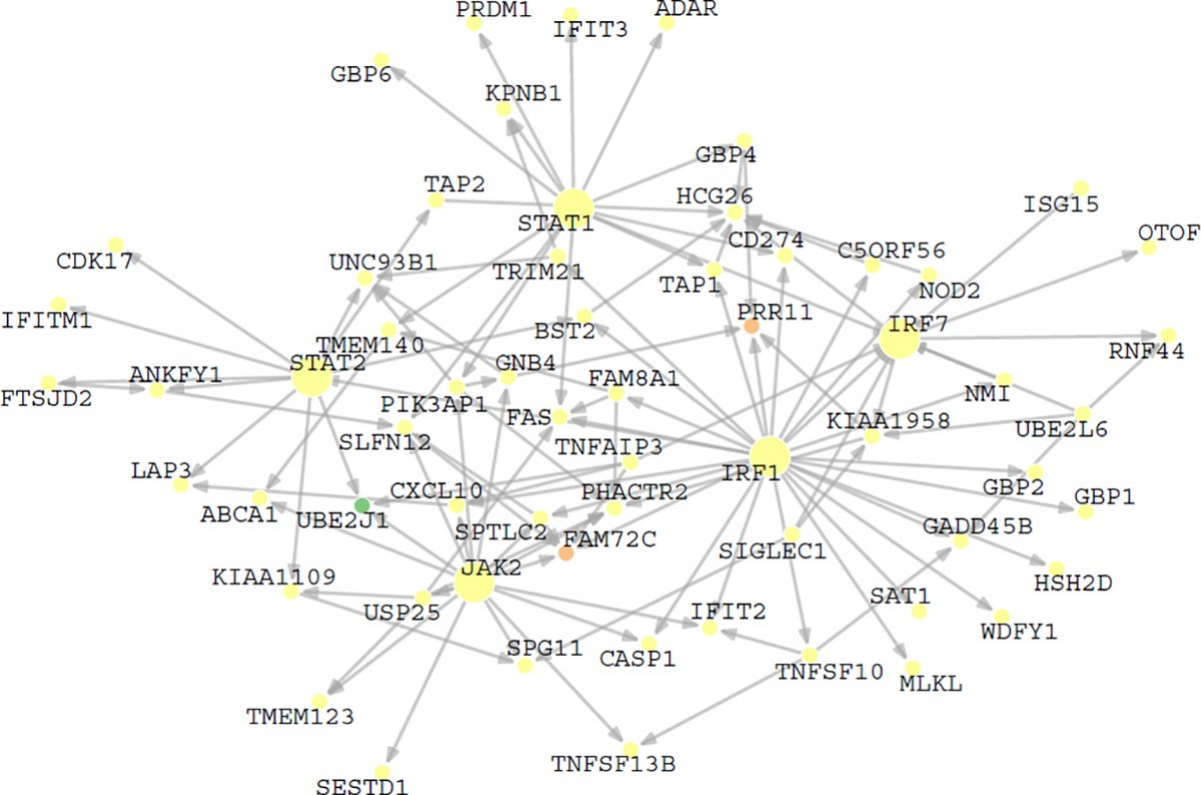
The subnetwork of *IFR1*, *IFR7*, *STAT1*, *STAT2*, *JAK2* and their neighbors. The color of a node represents one of the WGCNA modules, Yellow, Green or Tan.

Subsequently, we examined the GGIs among these JAK-STAT pathway genes and IFN regulatory genes, *STAT1*, *STAT2*, *JAK2*, *IRF1* and *IRF7*, and their first-degree neighbors (Fig 8). Some of our findings were consistent with the literature. For example, *IRF1* and *STAT1* can upregulate *IRF7* gene expression [26, 27]. *FAS* is downstream of many JAK-STAT pathway genes, indicating its crucial role in regulating cell death in SLE [28, 29]. Some known interactions were not found in the final network, e.g. the interactions between *STAT1*, *STAT2*, and *IRF1* [30], reflecting some limitations of the method and data. Nevertheless, the network successfully detected the central roles of JAK-STAT pathway in SLE, and many interactions may be novel and worth further validation.

**Fig 9:**
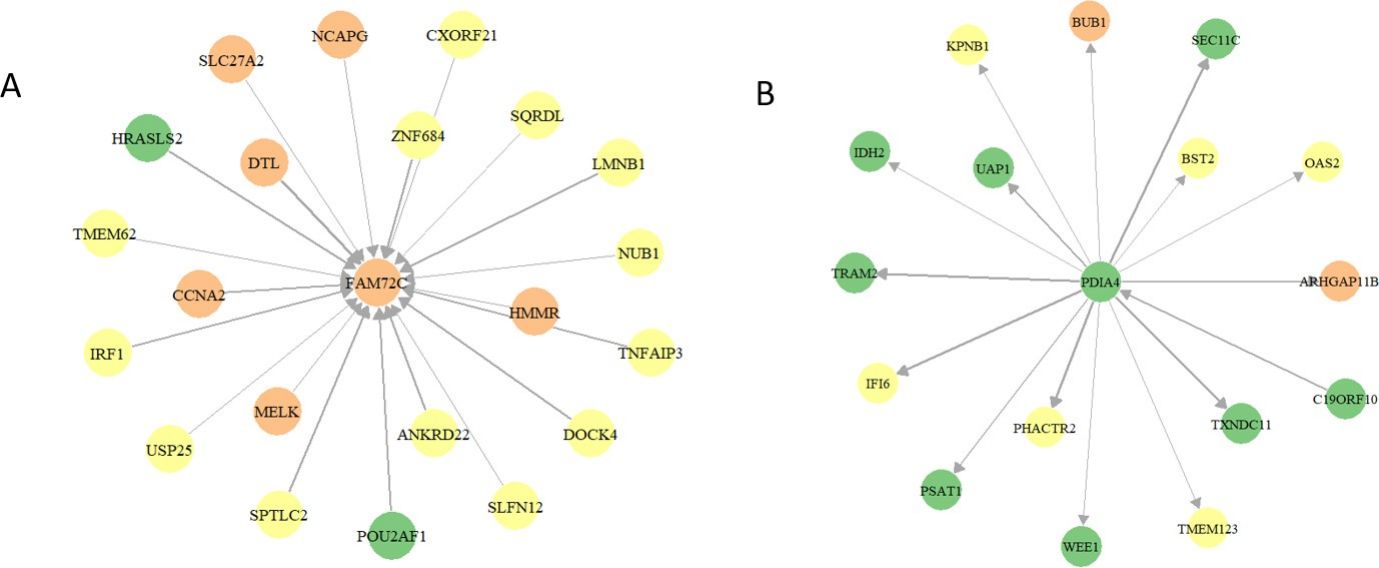
**The first-degree neighbors of (A) *FAM72C* and (B) *PDIA4* genes.** The color of a node represents one of the WGCNA modules, Yellow, Green or Tan, and the width of an edge corresponds to the edge weight, i.e. the edge frequency in the 100 runs.

Other identified hub genes in the final network could also play important roles in SLE. Some of them are still novel and further experiments may be needed to elucidate their roles in SLE. For example, *DHRS9* encodes a protein known as dehydrogenase/reductase (SDR family) member 9. In a recent study, evidence showed that the expression of *DHRS9* changed significantly with the addition of SLE immune complexes in peripheral blood mononuclear cells, indicating the potential regulatory role of this gene in SLE [31]. Notably, ten out of the top 12 hub genes were from the Yellow module, and the other two hubs genes are *FAM72C* and *PDIA4*, from the Tan and Green modules, respectively. *FAM72C* encodes a neuronal progenitor cell self-renewal supporting protein and is involved with cellular proliferation in cancerous cells [32], but its function in blood and SLE is unknown. The Tan module was enriched by many cell cycle related genes. In addition, many genes from the three modules ‘regulated’ *FAM72C* (Fig 9A). Therefore, we hypothesized that *FAM72C* may play a role when the INF and JAT-STAT pathways trigger the abnormal cell cycle of some peripheral cells, e.g. the abnormal activation of B cells in SLE [33]. In contrast, many genes, including some INF pathway genes, e.g. *OAS2*, *BST2*, *IFI6*, were ‘regulated by’ *PDIA4*, an endoplasmic reticulum-stress pathway gene (Fig 9B). Some studies found that endoplasmic reticulum-stress regulates some INF pathway genes [34, 35]. The interactions between *PDIA4* and INF pathway genes require further investigation.

### Key driver genes

We identified multiple key driver genes by integrating the network structure and differential expression analysis (Table 4). Differential expression analysis usually focuses on genes with relatively large fold changes to overcome the false positive issues. However, this approach could overlook some essential genes with small fold changes, especially when such genes are often located on the upstream side of a pathway, and activation of such genes could lead to downstream gene changes in the pathway (e.g. signal transduction). Upon binding of a chemical or physical signal to the receptor, a series of events will occur (e.g. protein phosphorylation), leading to a cell response [36]. In our analyses, we tried to fill in such gaps. If a large proportion of the neighbors of the target gene were significant differentially-expressed genes, such genes should be carefully studied even if the genes themselves were not significant differentially-expressed genes.

**Table 4:** Key diver genes.

Gene	Number of children	Average Z-score of children	Gene	Number of Markov blanket genes	Average Z-score of the Markov blanket
DHX58*	9	7.45	**TNFSF10**	42	6.52
USP18	7	7.11	**DHX58**	43	6.34
**TNFSF10**	8	6.92	**SIGLEC1**	55	6.33
UBE2L6	7	6.20	**JAK2**	59	5.90
**IRF1**	25	5.86	PNPT1	53	5.71
**STAT1**	13	5.62	**PML**	38	5.65
STAT2	10	5.61	**IRF1**	72	5.50
**JAK2**	15	5.36	**STAT1**	39	5.44
**SIGLEC1**	10	5.35	TNFAIP3	49	5.43
**PML**	8	5.30	IFI35	38	5.24

* The common genes in both lists are in bold.

We used two separate methods to define the neighbors that a key driver gene could affect. One was restricted to the ‘children’ and the other one was restricted to the ‘Markov blanket’. Usually, the Markov blanket could be viewed as the ‘maximum boundary’ of one gene effect. Therefore, the Markov blanket neighbor definition was relatively more liberal than the children definition. Table 4 lists the top 10 key driver genes for each definition.

Many of the key driver genes shared in both definitions, e.g. *TNFSF10*, *DHX58*, *SIGLEC1*, *JAK2*, *IRF1*, and *PML*. As discussed previously, *JAK2* and *IRF1* were critical for the IFN and JAK-STAT pathways. *TNFSF10* (*TRAIL*) encodes a tumor necrosis factor (ligand) superfamily member and is transcriptionally regulated by IRF1 [37]. Similarly, cytosolic nucleic acid sensors, including *DHX58* which is regulated by IRF1 [38], could stimulate type I IFN production and may serve as potential therapeutic targets for SLE [39, 40]. *SIGLEC1* was discovered as a biomarker of disease activity in SLE and could serve as a negative regulator of type I IFN production [41, 42]. It is known that *PML* transcription can be introduced by IFN, but it is unclear how PML mediates downstream signaling in SLE. [43, 44] In summary, the key driver-gene analysis suggested that JAK-STAT and IFN pathways played important roles in SLE. Furthermore, key driver-gene analysis is supplemental to hub gene analysis for gene prioritization. For example, *PDIA4* and *JAK2* have the same number of children (15), but the mean Z-score of the children of *JAK2* (5.36) is much higher than that of *PDIA4* (3.62). This indicates *JAK2* may have a higher functional impact than *PDIA4*.

## Discussion

In summary, we developed a novel way to build a Bayesian network based on transcriptomic data and literature mining for prior information. As an example, the method was implemented using transcriptomic data of pre-selected gene modules from SLE patients. Both hub gene and key driver-gene analyses suggested that the broad immunomodulatory effects mediated by the JAK-STAT pathway were critical for SLE. As a validation of our analysis, baricitinib, a JAK1/JAK2 inhibitor, showed success in a double-blind, randomized, placebo-control Phase 2 trial [4].

Despite interesting and novel findings, our methods have limitations. First, during the final network derivation, prior information was important. There are only limited ways that we could obtain gene-gene regulatory relations. We used text-mining because we believe it is the most comprehensive way to collect cumulatively all gene-gene regulatory relationships from potential all experiments conducted and assign confidence scores to control how that information can be used during the Bayesian network construction. Alternatively, curated databases with regulatory relationships, e.g. MetaBase (https://portal.genego.com/), RegNetwork [45], and TRRUST [46], can also be used as prior information. However, these databases are often biased due to the higher criteria for a regulatory relationship to enter the database, and the availability, accuracy, and update frequency highly depend on the research group maintaining the database. As a sensitivity analysis, we tested MetaBase as prior information. Interestingly, IFR1, STAT1, and STAT2 are among the top four hub genes. Although JAK2 was not identified as one of the top hub genes, the results indicated the essence of JAK-STAT pathway for SLE, consistent with that from using I2E text-mining as prior. Additionally, new GGI results could be incorporated into prior information, and a new network derived reiteratively. This would help to incorporate all different layers of data dynamically and could be viewed as a multivariate version of ‘meta-analysis’. Second, although evidence has suggested that blood was a reasonable tissue to profile the disease, a debate may arise whether whole blood is the best tissue to characterize SLE [47]. Additionally, as blood is a mixture of different cell types, there is a lack of single-cell transcriptome profiling. Therefore, our network may not reflect GGI for a particular cell type, but an average of the mixed cell types. Third, due to the computational limitations, only a subset of genes was included and GGIs of some relevant genes (e.g. *JAK1*) could not be inferred. Last but not least, a Bayesian network could not accommodate feedbacks or loops, which are critical components for biological mechanisms. Despite these limitations, our network can still provide valuable information for scientists to understand SLE. Additionally, the methods of constructing a Bayesian network and identifying key driver genes could be easily applicable to other diseases with a suitable dataset.

## Supporting information

S1 Table The genes in the three selected WGCNA modules. The table lists the modules (Module), transcript clusters (TC), gene symbol (geneSymbol) and gene name (geneName).
(XLSX)

S2 Table Prior information from text-mining. Each row corresponds one regulatory relationship (“Gene A”, “Relation”, “Gene B”), PubMed ID, and the original text describing the relationship (Hit).
(XLSX)

S3 Table The prior network edges with weight. (XLSX)

S4 Table The final network edges. Color represents the WGCNA module name and weight is the edge frequency in 100 runs.
(XLSX)
